# Widespread association of ERα with RMRP and tRNA genes in MCF-7 cells and breast cancers

**DOI:** 10.1016/j.gene.2022.146280

**Published:** 2022-05-05

**Authors:** Jodie R. Malcolm, Natasha K. Leese, Philippa I. Lamond-Warner, William J. Brackenbury, Robert J. White

**Affiliations:** Department of Biology, The University of York, Heslington Road, YO10 5DD, United Kingdom

**Keywords:** tRNA, Breast Cancer, Estrogen Receptor, RMRP, RNA Polymerase III, 7SL, **Erα**, Estrogen Receptor alpha, **ERE**, Estrogen Responsive Element, **Pol III**, RNA Polymerase III, **TBP**, TATA-Binding Protein, **SERM**, Selective Estrogen Receptor Modulator, **SERD**, Selective Estrogen Receptor Down-regulator, **HAT**, Histone Acetyltransferase, **GEO**, Gene Expression Omnibus, **IDC**, Invasive Ductal Carcinoma, **SRA**, Sequence Read Archive, **BC**, Breast Cancer, **GO**, Good Outcome, **PO**, Poor Outcome, **Met**, Metastasis

## Abstract

tRNA gene transcription by RNA polymerase III (Pol III) is a tightly regulated process, but dysregulated Pol III transcription is widely observed in cancers. Approximately 75% of all breast cancers are positive for expression of Estrogen Receptor alpha (ERα), which acts as a key driver of disease. MCF-7 cells rapidly upregulate tRNA gene transcription in response to estrogen and ChIP-PCR demonstrated ERα enrichment at *tRNA^Leu^* and *5S rRNA* genes in this breast cancer cell line. While these data implicate the ERα as a Pol III transcriptional regulator, how widespread this regulation is across the 631 tRNA genes has yet to be revealed. Through analyses of ERα ChIP-seq datasets, we show that ERα interacts with hundreds of tRNA genes, not only in MCF-7 cells, but also in primary human breast tumours and distant metastases. The extent of ERα association with tRNA genes varies between breast cancer cell lines and does not correlate with levels of ERα binding to its canonical target gene *GREB1*. Amongst other Pol III-transcribed genes, ERα is consistently enriched at the long non-coding RNA gene *RMRP*, a positive regulator of cell cycle progression that is subject to focal amplification in tumours. Another Pol III template targeted by ERα is the *RN7SL1* gene, which is strongly implicated in breast cancer pathology by inducing inflammatory responses in tumours. Our data indicate that Pol III-transcribed non-coding genes should be added to the list of ERα targets in breast cancer.

## Highlights

• Estrogen receptor (ER) interacts with hundreds of tRNA genes (tDNAs) in MCF-7 cells.
• Hundreds of tDNAs are also targeted in primary breast tumours and metastases.
• Canonical estrogen response element is not found near top tDNA targets of ER.
• ER also targets non-coding breast cancer driver gene *RMRP.*
• ER also targets *RN7SL1* gene that promotes breast cancer progression.

## 1 Introduction

Transcription of the human nuclear genome is mediated by three specialised RNA polymerase enzymes, each of which is responsible for expressing a specific category of genes (Cramer, 2002, Vannini and Cramer, 2012). RNA polymerase III (Pol III) is a complex of 17 subunits, making it the largest of the three nuclear RNA polymerase enzymes (Vannini and Cramer, 2012). Pol III is required for the synthesis of many non-coding RNAs, the most abundant of which are 5S rRNA, tRNA and 7SL RNA (Dieci et al., 2007). Recruitment of Pol III to its target genes requires TFIIIB, which is in most cases recruited by TFIIIC, a six-subunit complex that binds two internal promoter motifs, termed the A and B boxes, downstream of the transcription start site of tRNA genes (Ramsay and Vannini, 2018, Schramm and Hernandez, 2002).

Dysregulated transcription by Pol III has been widely observed in many types of tumour, including ovarian and breast cancers (Krishnan et al., 2016, Pavon-Eternod et al., 2009, White, 2004, Winter et al., 2000, Zhang et al., 2018). Furthermore, several Pol III-specific products have been implicated in promoting disease progression, such as RMRP, BC200, 7SL and specific tRNAs (Goodarzi et al., 2016, Nabet et al., 2017, Rheinbay et al., 2020). In 2020, 2.2 million cases of breast cancer were diagnosed around the world (Sung et al., 2021). Estrogen Receptor (ERα) acts as a potent driver of disease for approximately 75% of all breast cancers. The current standards of care for ERα + breast cancer include the selective estrogen receptor modulator (SERM) tamoxifen or the selective estrogen receptor down regulator (SERD) fulvestrant, and a combination of additional targeted agents, such as monoclonal antibodies or cyclin-dependent kinase inhibitors (Johnston and Cheung, 2018). Despite initial positive outcomes observed for many ERα + breast cancers, between 40 and 50% of patients receiving endocrine therapy acquire resistance after five or more years of treatment, ultimately leading to relapse, metastatic disease and death (Anurag et al., 2018). This highlights the importance of further understanding the action of ERα in driving disease, in order to design potent therapeutics that overcome this acquired resistance to endocrine therapy.

ERα is a nuclear receptor that specifically coordinates transcriptional changes for many target genes in response to estradiol. Its paradigm mechanism of gene induction involves an estrogen-responsive element (ERE), which is a palindromic consensus sequence of two half site motifs separated by a 3 bp spacer (5′-GGTCAnnnTGACC-3′); they are found at enhancer and promoter regions of many ERα-regulated genes that enable the hormone receptor to directly bind to the gene via its DNA-binding domain and bring about transcription by recruitment of additional transcription factors, such as histone acetyltransferases. An alternative, less-characterized mechanism involves ERα tethering to other transcription factors that are themselves DNA-bound and act to either stabilize transcription complexes, or recruit additional cofactors; in this instance, the ERα itself is not directly bound to the DNA.

Stimulation of MCF-7 breast cancer cells with estrogen induces rapid and profound transcriptional changes, in which many tRNA genes are affected (Hah et al., 2011). Furthermore, the ERα was found to amplify alcohol-induced deregulation of 5S rRNA and tRNALeu genes in MCF-7 cells, whereas tamoxifen repressed these Pol III targets (Zhang et al., 2013, Zhong et al., 2014). Thus, ERα is implicated in the control of Pol III transcription in MCF-7 cells.

To determine how extensively ERα targets Pol III-transcribed genes, ERα ChIP-seq datasets from ENCODE, the NCBI SRA and Gene Expression Omnibus (GEO) were analysed. This revealed widespread interaction of ERα with many tRNA genes in MCF-7 cells and in ERα + ve patient breast cancer samples, as well as in distant breast cancer metastases. However, minimal interaction with tRNA genes was detected in ERα-negative MDA-MB-231 cells with a stably-integrated ERα expression vector, despite robust binding to established targets with consensus ERE sequence motifs. These data suggest that recruitment of ERα to tRNA genes may depend on unknown factors present in MCF-7 and ERα + human breast cancer cells, but absent from the MDA-MB-231 cell line. Possibilities include specific pioneer factors and/or proteins involved in chromatin regulation or ERα tethering. Two other pol III-transcribed loci that attract ERα in breast cancer cells are the *RMRP* gene, which has been implicated by recurrent mutation as an oncogenic driver (Rheinbay et al., 2020, Rheinbay et al., 2017), and the RN7SL1 gene, which has been shown to promote inflammation and progression of breast tumours (Nabet et al., 2017).

## 2 Materials & methods

### 2.1 ERα ChIP-seq data from ENCODE

Filtered alignments for ERα ChIP-seq experiments carried out in genetically modified human MCF-7 cell lines (*insertion, using CRISPR to generate an MCF-7 cell line stably expressing a C-terminal LAP-tag containing eGFP, fused to ESR1*) were downloaded in Binary Alignment Map (BAM) file format from ENCODE (available at https://www.encodeproject.org/; Table 1.) The ChIP-seq experiment was carried out in the lab of Michael Snyder, Stanford, and released to the open-access ENCODE database on July 8th 2020 (Davis et al., 2018, ENCODE Project Consortium, 2012).

**Table 1:** ENCODE accession number and experimental file information.

Experiment	BAM file	Target	Tissue Type	Genome Assembly
ENCSR463GOT	ENCFF365BIT	ERα	Human MCF-7 cell line	GRCh38
	ENCFF063JMY			

### 2.2 MCF-7 and clinical breast cancer ERα ChIP-seq data

ERα ChIP-seq was performed in patient samples and in the MCF-7 and ZR-75–1 cell lines (Ross-Innes et al., 2012). Datasets were deposited into the National Centre for Biotechnology Information (NCBI) Sequence Read Archive (SRA) Run Selector under the accession number of PRJNA147213 (Table 2) (Leinonen et al., 2011). SRA files were obtained and converted to *FastQ* file format and then to EaSeq-readable BAM file format using Galaxy (version 21.05.rc1) “Genomic File Manipulation” tools.

**Table 2:** SRA experimental file information for PRJNA147213 ChIP-seq datasets.

Experiment	SRA File	Tissue Type	Target	Genome Assembly
PRJNA147213	SRR1021749	Invasive Ductal Carcinoma	ERα	NCBI36 (hg18)
	SRR1021750			
	SRR1021756			
	SRR1021758			
	SRR1021788	MCF-7 cell line		
	SRR1021790	ZR-75–1 cell line		
	SRR1021765	Metastases		
	SRR1021766			
	SRR1021767			

### 2.3 MDA-MB-231 ERα ChIP-seq data

ChIP-seq was performed in the ERα -ve MDA-MB-231 cell line that was stably expressing a wild type ERα construct (Stender et al., 2010) and the dataset was deposited into the NCBI Gene Expression Omnibus (GEO) under the accession number PRJNA129093 (Table 3). Wild type ERα ChIP-seq data were downloaded in Bed format.

**Table 3:** Experimental file information for ERα -ve MDA-MB-231 dataset.

Experiment	File	Target	Cell line	Genome Assembly
PRJNA129093	GSM560853	ERα	MDA-MB-231	NCBI36 (hg18)

### 2.4 EaSeq for the quantification of ERα signals at tRNA genes

BAM or BED files containing ERα ChIP-Seq replicates were uploaded into EaSeq “DataSets”. Complete tables of tracked GRCh38 (hg38) and NCBI36 (hg18) tRNA genes were downloaded from the UCSC Table Browser, (available at https://genome.ucsc.edu) and imported as EaSeq “regionsets” to be used as regions of interest.

**Fig. 1:**
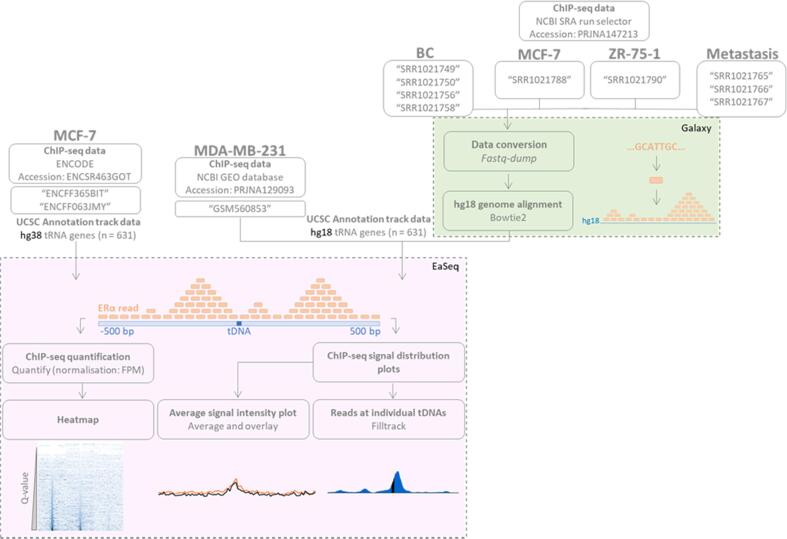
**Overview of bioinformatic pipeline for ERα ChIP-Seq analysis and visualisation at tRNA genes.** ERα ChIP-seq analysis performed in MCF-7 cells, ZR-75 cells (ENCODE and NCBI SRA), MDA-MB-231 cells (NCBI GEO) and patient breast cancer (BC) tumour samples and distant metastases (NCBI SRA) were analysed using EaSeq, as illustrated in the purple box. tRNA track annotations were obtained from the UCSC (hg38 for MCF-7 and hg18 for MDA-MB-231, MCF-7, ZR-75–1, metastases and BC datasets). PRJNA147213 SRA files were first converted to EaSeq-readable BAM file format using Galaxy, as illustrated by the green box. (For interpretation of the references to colour in this figure legend, the reader is referred to the web version of this article.)

To quantify ERα peaks at tRNA genes, the EaSeq “quantify” tool was used. This tool counts the number of reads from the “DataSet” that overlaps with the specified regions of interest in the “Regionset”. For this process, default settings of “normalize to reads per million” and “normalize counts to DNA fragments” were left checked. The default setting of “normalize signal to a size of 1000 bp” was unchecked. Quantification analyses were performed at ± 500 bp from the start of tRNA genes. This generated quantification values that are referred to as “Q-values”. Following quantification, all of the tRNAs were sorted in order of increasing Q-value. Data visualisation was performed using EaSeq “heatmap”, “average signal intensity plot” and “filltrack” tools (Fig. 1). EaSeq is available at http://easeq.net (Lerdrup et al., 2016).

### 2.5 Classification or ERα-bound tRNA genes based on association with proliferation or differentiation

tRNA pools from several cell types were categorised previously based on their induction in the cellular processes of proliferation, differentiation or other (Gingold et al., 2014). The top 50 ERα-bound tRNA genes (highest Q-values) confirmed in the ENCODE ERα MCF-7 ChIP-seq data analysis described above were independently compared against these tRNA gene categories.

### 2.6 Motif analysis

Motif investigations were conducted in tDNA genes which had the largest ERα Q-values following ChIP-seq analysis in the MCF-7 dataset (n = 19 for ENCODE data). The tRNA genomic coordinates were obtained (FASTA format, ± 20 kbp) using the NCBI Gene database and supplied to the Multiple EM for Motif Elicitation ChIP (MEME) Suite (v 5.3.0) to search for known sequences, using default settings. Sequence motifs of the conserved A and B box and of the ERE and half EREs were supplied to the program.

### 2.7 tRNA gene coordinate remapping

To determine the overlap between the tRNA genes in which ERα strongly associates in the hg38 MCF-7 cells and the hg18 breast cancer samples, the NCBI genome remapping service (available at https://www.ncbi.nlm.nih.gov/genome/tools/remap) was used to retrospectively remap the hg38 tRNA gene coordinates to the NCBI36 hg18 gene coordinates. Of the 631 hg38 tRNA gene coordinates provided, 80% of the tRNA genes were successfully remapped.

### 2.8 Chromatin immunoprecipitation and quantitative polymerase chain reaction (ChIP-qPCR)

MCF-7 cells (ECACC; 18F024) were grown in DMEM (Gibco; 41966–029) with 10% FBS (Gibco; 102790–098) and 1% Penicillin Streptomycin (Gibco; 15070–063). Cells were crosslinked with 1% formaldehyde for 8 min at room temperature. Crosslinking was inhibited by adding equal volume of ice cold quenching buffer (250 mM Glycine/ 2 mM EDTA / TBS) to the cells, followed by centrifugation (1,100xg, 5 min, 4℃). This step was repeated for a total of two washes in the quenching buffer. MCF-7 cells were then lysed in Lysis Buffer 1 (50 mM Hepes-KOH [pH 7.5], 140 mM NaCl, 1 mM EDTA, 10% glycerol, 0.5% NP-40, 0.25% Triton X-100) for 10 min at 4℃. Cell lysate was collected by centrifugation (1,100xg, 5 min, 4℃) and resuspended in Lysis Buffer 2 (10 mM Tris-HCl [pH8.0], 200 mM EDTA, 1 mM EGTA) for 10 min at 4℃. MCF-7 nuclei were harvested by centrifugation (1,100xg, 5 min, 4℃) and resuspended in Lysis Buffer 3 (10 mM Tris-HCl [pH 8.0], 100 mM NaCl, 1 mM EDTA, 0.5 mM EGTA, 0.1% sodium deoxycholate, 0.5 % N-lauroylsarcosine), before sonication (Bioruptor; 30 s per sonication cycle for a total of 12 sonication cycles). 0.1% of Triton X-100 was added to nuclear extract and debris was pelleted by centrifugation (5,000 xg, 10 min, 4℃).

Antiserum 1901 was raised by immunizing rabbits with synthetic peptide PKRPLTFDTNEFHIPLVT (residues 1374–1391 of the RPC155 subunit of human pol III), coupled to KLH. For immunoprecipitation, protein A Dynabeads were washed in blocking solution (0.5% BSA in PBS). Dynabeads were next incubated in blocking solution containing either 15 μl pre-immune serum (PI), 15 μl anti-pol III serum (1901) or 6 μg of anti-ERα antibody (Abcam; ab32063) for 2 h at 4℃. Protein A:antibody-containing beads were then added to nuclear lysate and mixed overnight at 4℃. Next, the Dynabeads were collected on a magnetic rack and washed in RIPA buffer (50 mM Hepes-KOH [pH 7.6], 500 mM LiCl, 1 mM EDTA, 1% NP-40, 0.7% Na-Deoxycholate) for a total of 6 washes (5 min per wash, 4℃). The sample-containing beads were then washed with 50 mM NaCl Tris EDTA buffer for a total of 2 washes (5 min per wash, 4℃) before elution with elution buffer (100 mM NaHCO3 + 1% SDS) at 50℃ for 10 min. Crosslinking was then reversed by heating at 55℃, overnight. To collect chromatin, 1.8x SPRY beads were added to the samples and collected on a magnetic stand after 10 min. Beads were washed twice with 80% ethanol. DNA was then eluted from the beads by resuspending in dH_2_O and heating for 5 min at 65℃, for a total of two elution steps. Eluates were then pooled together.

Real time quantitative PCR (qPCR) was performed using 2 ng of chromatin extraction (input) and 1 μl of ChIP sample per reaction on a QuantStudio™ 3 qPCR system (Thermo Fisher). Triplicate reactions of each sample were performed using LUNA Universal qPCR Master Mix (NEB; M3003L) on 96 well plates. The cycling parameters are shown in Table 4. Primers used for the amplification of pol III products are shown in Table 5.

**Table 4:** qPCR Cycling Parameters. qPCR reactions were carried out for a total of 42 cycles.

Temperature (℃)	Time (mm:ss)	
50	02:00	
95	03:00	
95	00:20	X 42
62	00:15	
72	00:15

**Table 5:** Primer sequences for qPCR amplification.

Gene Target	Forward	Reverse
*tRNA Pro-TGG-1*–*1*	TTCTGGCTCGTTGGTCTAG	AGGGGCTCGTCCGG
*tRNA Arg-CCG-2*–*1*	GTGGCCTAATGGATAAGGCATCA	CTAATCTCACGCGACCCAGATG
*tRNA Met-CAT-1*–*1*	ACTAGGTGCCTCGTTAGCGCAG	ACAAAATTATTGTGCCCCGTGTGAGG
*tRNA Leu-AAG-2*–*4*	CATATTGCAGCTGGGTAGCG	CCGAAGAGACTGGAGCCTTA
*RMRP*	AAGAAGCGTATCCCGCTGAG	GCACTGCCTGCGTAACTAGA
*RN7SL1*	TATCCGACCGCCGGGC	AGTGGCTATTCACAGGCGCG

## 3 Results

### 3.1 Widespread localisation of ERα at tRNA genes in MCF-7 cells

**Fig. 2:**
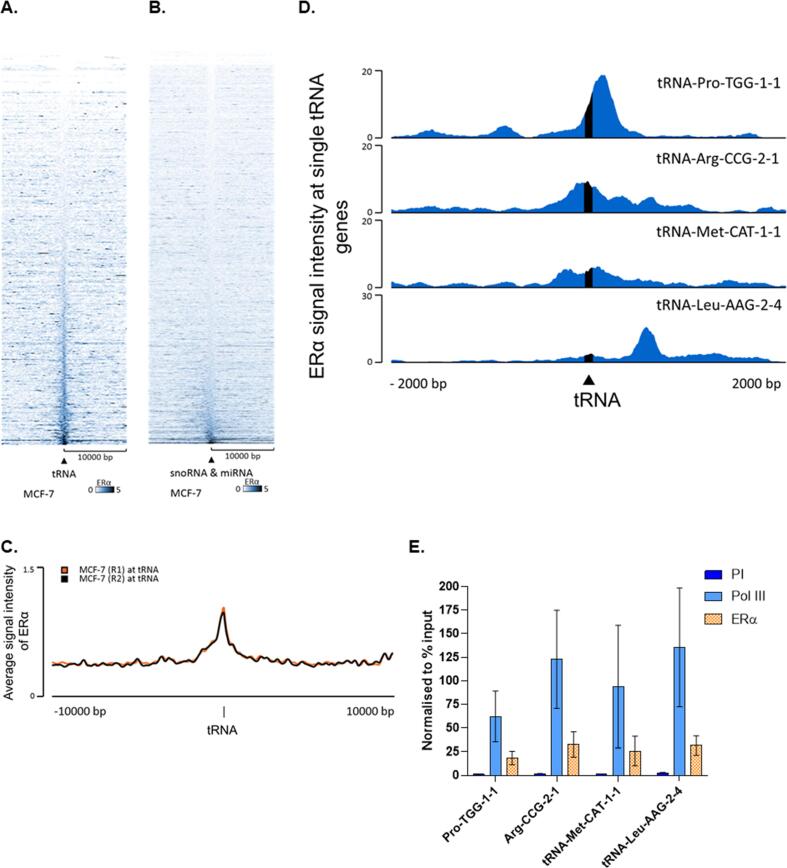
**ERα is associated with many tRNA genes in MCF-7 cells.** (A) Heatmap of ERα binding events across hg38 tRNA genes in the MCF-7 cell line. Window represents the ± 10 kb region from the centre of tRNA genes. Genes arranged in order of increasing Q-value. (B) Heatmap of ERα binding events across hg38 snoRNA and miRNA genes in the MCF-7 cell line. Window represents the ± 10 kb region from the centre of snoRNA and miRNA genes. Genes arranged in order of increasing Q-value. (C) Average signal intensity overlay of two ERα ChIP-seq replicates (R1 and R2) across all tRNA genes in the MCF-7 cell line. Window represents the ± 10 kb region from the centre of tRNA genes. (D) Representative filltrack images of ERα binding at individual tRNA genes (tRNA-Pro-TGG-1–1, tRNA-Arg-CCG-2–1, tRNA-Met-CAT-1–1, and tRNA-Leu-AAG-2–4). (E) ChIP-qPCR assays carried out at individual tRNA genes (tRNA-Pro-TGG-1–1, tRNA-Arg-CCG-2–1, tRNA-Met-CAT-1–1,and tRNA-Leu-AAG-2–4) to confirm pol III and ERα enrichment. N = 3. Error bars are ± SEM. qPCR values have been normalised to % input.

GRO-seq analysis showed that MCF-7 cells rapidly increase production of many tRNAs in response to estradiol stimulation (Hah et al., 2011). To investigate how extensively ERα targets the 631 tRNA genes annotated in the human genome, we interrogated ChIP-seq datasets from two replicate experiments. This revealed widespread interaction of ERα with>300 tRNA genes in MCF-7 cells (Fig. 2A; Supplementary Fig. S1). A heatmap of binding reveals clear concentration of ERα at about half of all tRNA genes, relative to the 10 kb upstream and downstream flanking regions. This striking observation was confirmed using an independent orthogonal MCF-7 data set, as well as in ZR-75–1 cells, a second ERα + breast cancer model (Supplementary Fig. S1). The specificity of this association is demonstrated by comparison with genes for snoRNAs and miRNAs, two other classes of short non-coding RNA, where only a very small minority of loci interact with ERα (Fig. 2B). Average signal intensity plots confirm peaks of ERα binding that overlap with the tRNA genes (Fig. 2C), which can also be seen by plotting the reads at individual tDNAs, such as the *tRNA-Pro-TGG-1*–*1*, *tRNA-Arg-CCG-2*–*1* and *tRNA-Met-CAT-1*–*1* genes (Fig. 2D). Examples were also found of binding nearby rather than at a particular tDNA, e.g. the *tRNA-Leu-AAG-2*–*4* gene. Pol III and ERα enrichment at the tRNA genes in MCF-7 cells have been confirmed through ChIP-qPCR analysis, where ERα binding is enriched relative to the pre-immune control (Fig. 2E). This finding supports independently the ChIP-seq evidence that ERα binds to tRNA genes in MCF-7 cells.

### 3.2 Relationship of ERα-targeted tRNA genes with groups implicated in proliferation or differentiation

**Fig. 3:**
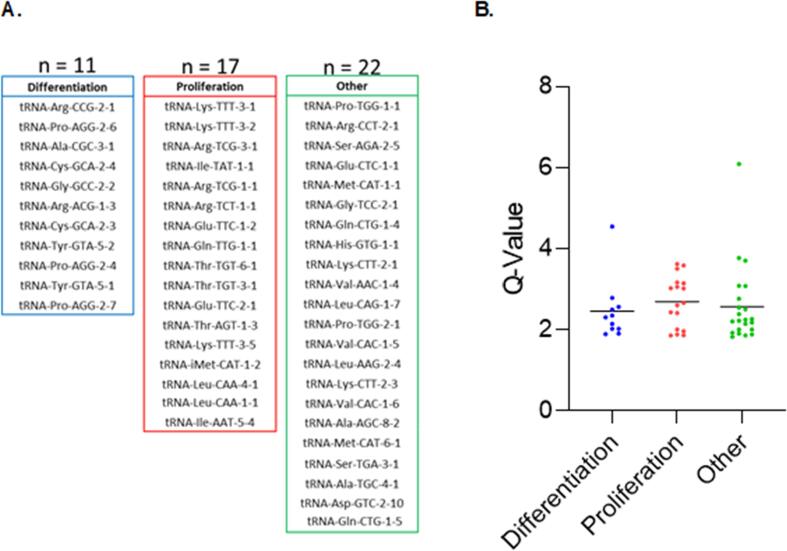
**Categories of tRNA genes targeted by ERα in MCF-7 cells** (A) Classification of top fifty ERα-bound tRNA genes in MCF-7 cells according to the categories proposed by Gingold et al. (2014) as preferentially associated with differentiation (blue), proliferation (red) or other cellular processes (green). (B) Quantification of ERα binding at the top fifty ERα-bound tRNA genes based on their preferential association with differentiation, proliferation, or other cellular processes.

Previous analysis of tRNA expression identified transcripts that are induced preferentially during cell differentiation and a distinct group that is expressed preferentially in proliferating cell types, including bladder, colon and prostate cancers (Gingold et al., 2014). Although breast cancers were not included in those data, we analysed whether the tRNA targets of ERα identified by ChIP-seq in MCF-7 cells are enriched in either the differentiation or proliferation categories. We found that the top fifty ERα-bound tRNA genes (Supplementary Table ST1) are slightly depleted in the differentiation category relative to the proliferation group, although the difference is not statistically significant (Fig. 3A). Similarly, Q-values for binding are not significantly different (Fig. 3B), even after removal of outliers (Supplementary Fig. S2). Our data, therefore, do not provide clear evidence that ERα recruitment discriminates in MCF-7 cells between the previously suggested tRNA categories.

### 3.3 Consensus ERE and half ERE motifs are not required for recruitment of ERα to target tRNA genes

**Fig. 4:**
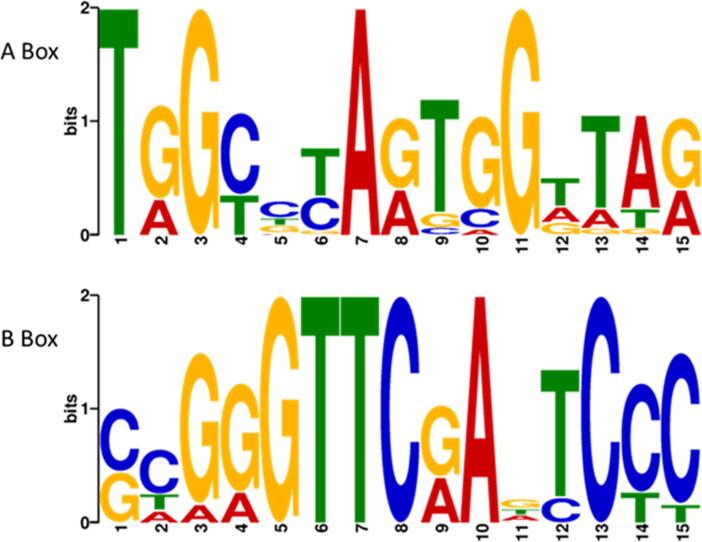
**Recruitment of ERα to tRNA genes does not require local consensus ERE motifs.** Position Weight Matrices (PWMs) of A and B box promoter sequences identified within the top 19 ERα-bound tRNA genes, but consensus ERE motifs were not detected.

At least two distinct mechanisms have been shown to recruit ERα to enhancer and promoter regions that regulate protein-coding genes: i) direct binding to a DNA sequence motif (ERE) via its DNA-binding domain; ii) protein/protein interactions with other DNA-binding transcription factors, such that ERα is not bound to the DNA directly. To investigate the predominant mechanism directing the ERα to tRNA genes, motif analysis was conducted to search for the full ERE or half ERE consensus elements around the tRNA genes that have the strongest ERα binding events in MCF-7 cells. The search included 20 kb up- and downstream of the target genes themselves. Motif analysis conducted in MEME-ChIP failed to identify either a full or half ERE in these regions; this was not due to technical failure, as the conserved A and B box promoter sequences were strongly enriched in all tRNA genes, as expected (Fig. 4). This does not exclude the possibility that non-canonical ERE sequences may be involved in ERα recruitment to tRNA genes. Furthermore, ERα may bind directly to ERE sites located more distally than 20 kb away and then associate with tRNA genes through looping of intervening chromatin. However, direct recognition of proximal consensus ERE sequences seems not to be required for recruiting the hormone receptor to the tRNA genes studied.

### 3.4 Minimal recruitment of exogenous ERα to tRNA genes in MDA-MB-231 cells

**Fig. 5:**
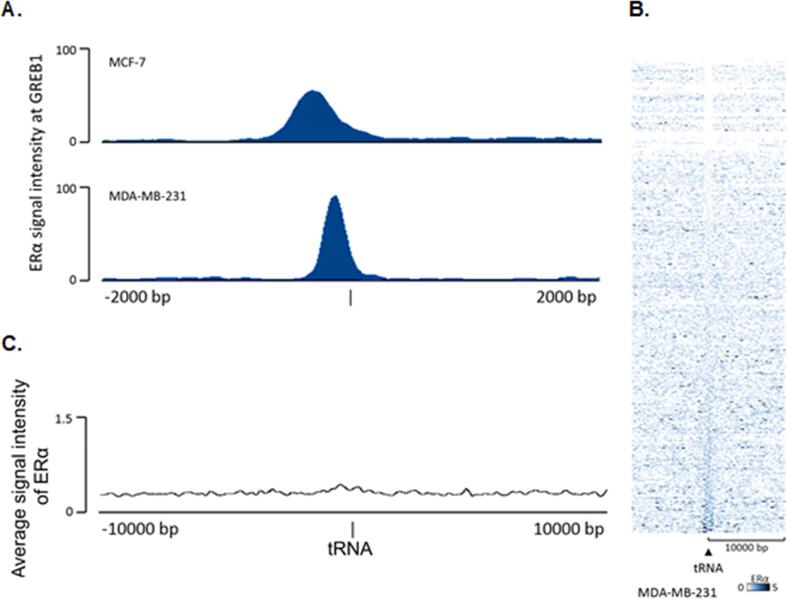
**Minimal interaction of exogenous ERα at tRNA genes in MDA-MB-231 cells.** (A) Binding of ERα in a ± 2 kb region upstream of the *GREB1* start site in MCF-7 cells (top) and MDA-MB-231 cells (bottom). (B) Heatmap of ERα binding events across hg18 tRNA genes in the MDA-MB-231 cell line. Window represents the ± 10 kb region from the centre of tRNA genes. Genes arranged in order of increasing Q-value. (C) Average signal intensity of ERα ChIP-seq across all tRNA genes in the MDA-MB-231 cell line. Window represents the ± 10 kb region from the centre of tRNA genes.

If recruitment of ERα to tRNA genes requires interactions with other protein(s) rather than direct recognition of DNA, it might vary between cell types according to availability of the necessary factor(s) and/or chromatin accessibility. To begin to explore this possibility, we investigated binding by exogenous ERα stably transfected into the MDA-MB-231 breast cancer cell line that lacks endogenous ERα (Stender et al., 2010). Binding by ERα upstream of the *GREB1* gene was at least as strong in transfected MDA-MB-231 cells as in MCF-7 cells (Fig. 5A). However, ChIP-seq revealed minimal interaction of the exogenous ERα with tRNA genes, as shown by heat map (Fig. 5B; Supplementary Fig. S1) and average signal intensity (Fig. 5C). This striking result has more than one potential explanation, but suggests that association of ERα with tRNA genes requires something absent from MDA-MB-231 cells, such as post-translational modifications and/or factors that influence access or retention.

### 3.5 Widespread association of ERα with tRNA genes in human tumours

**Fig. 6:**
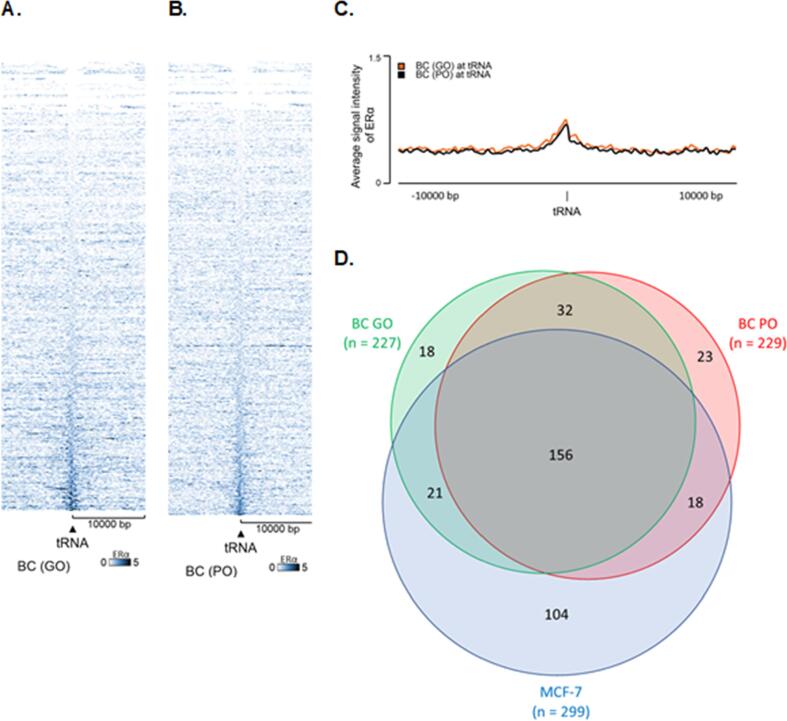
**ERα associates with many tRNA genes in primary breast tumours.** Heatmap of ERα binding events across hg18 tRNA genes in breast cancer (BC) samples from patients with good outcome (GO) (A) or poor outcome (PO) (B). Window represents the ± 10 kb region from the centre of tRNA genes. (C) Average ERα signal intensity overlay of GO and PO samples across all tRNA genes in the BC samples. Window represents the ± 10 kb region from the centre of tRNA genes. (D) Venn diagram representing the overlap in ERα tRNA binding events between the MCF-7 cell line and good outcome (GO) or poor outcome (PO) breast cancer (BC) datasets. Overlap correlates to binding events that obtained a Q-value > 0.5, following hg38 tRNA gene coordinate remapping.

ChIP-seq data are publicly available that analyse genome-wide binding of ERα in primary breast tumour samples from patients with invasive ductal carcinomas (Ross-Innes et al., 2012). Heatmaps of data from four patients again demonstrate that ERα associates with many tRNA genes, thereby establishing that our observations in MCF-7 cells reflect the situation in human tissue (Fig. 6A,B; Supplementary Fig. S1). Plots of average signal intensity confirmed peaks of ERα binding at the tRNA genes, relative to 20 kb of upstream and downstream surrounding genomic regions (Fig. 6C). Based on their subsequent survival, two of these patients were classified as having a good outcome (GO) and two as poor outcome (PO), but little difference was apparent between these in terms of numbers of tDNAs bound or average signal intensity (Fig. 6A-C). Substantial overlap was found between the sets of tDNAs bound by ERα in the GO and PO clinical samples, whereas MCF-7 cells showed more divergence (Fig. 6D). Adaptation of MCF-7 cells for sustained culture might explain, at least in part, the variation with respect to natural tumours. We conclude that large numbers of tRNA genes are targeted by endogenous ERα in primary breast cancers.

**Fig. 7:**
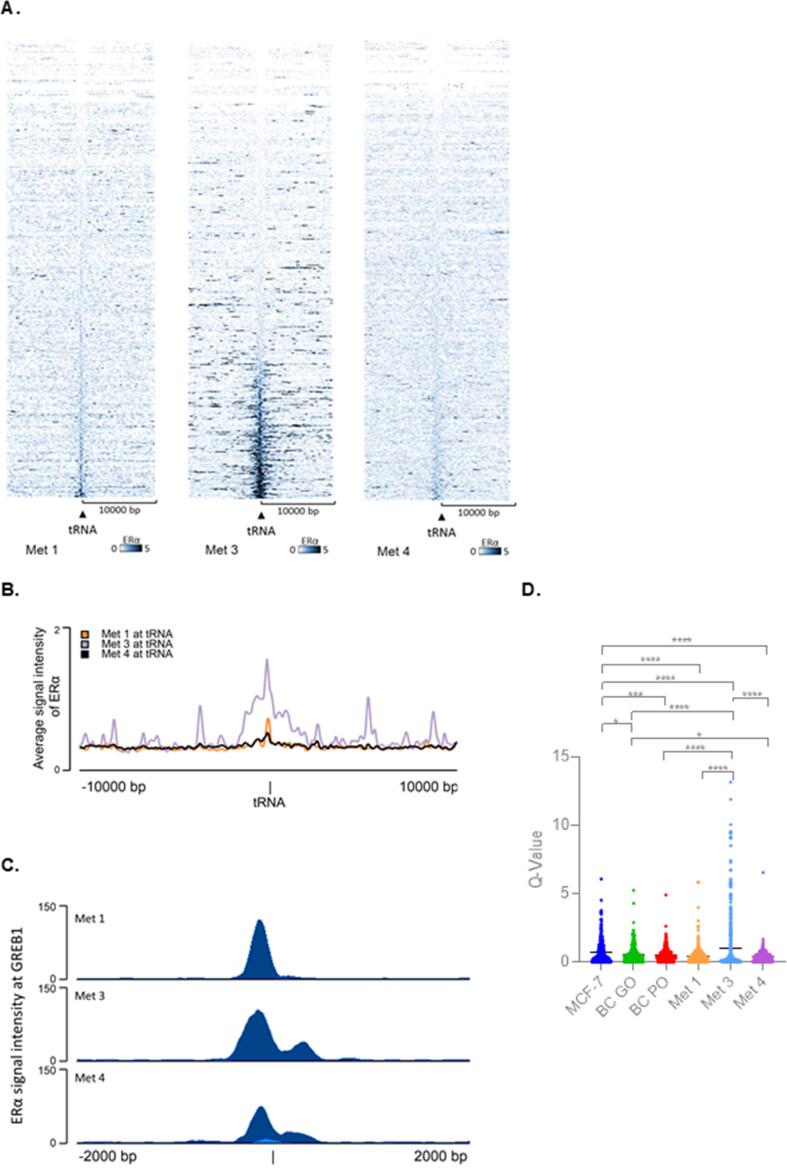
**ERα associates with many tRNA genes in breast cancer metastases.** (A) Heatmap of ERα binding events across hg18 tRNA genes in metastases from three patients. Window represents the ± 10 kb region from the centre of tRNA genes. (B) Average ERα signal intensity overlay across all tRNA genes in the three metastases. Window represents the ± 10 kb region from the centre of tRNA genes. (C) Binding of ERα in a ± 2 kb region upstream of the *GREB1* start site in metastatic samples.(D) Box and whisker plot showing Q-values of ERα binding at tDNAs in MCF-7 cells, GO and PO primary BC tumours and three distant metastases. * p = < 0.05; *** p = < 0.001; **** p = > 0.0001.

Biopsy samples of distant metastases were also analysed from three patients. These again showed clear accumulations of ERα at many tRNA genes, relative to the 10 kb upstream and downstream flanking regions, as revealed in heatmaps (Fig. 7A; Supplementary Fig. S1) and plots of average signal intensity (Fig. 7B). The metastatic sample from one patient (Met 3) displayed substantially stronger tDNA binding than the others. To assess whether this metastasis has ERα activity that is abnormally elevated, we compared binding to the well-characterized target GREB1 (Fig. 7C), which did not show a substantial increase in chromatin binding by ERα in Met 3. The data suggest that ERα interacts more strongly and selectively with a subset of tDNAs in this particular metastasis. This is clearly illustrated in Fig. 7D, which reveals that multiple tDNAs are targeted by ERα in Met 3 with strengths that are not seen in the other metastases, primary tumours or MCF-7 cells. The increase in association of ERα with tDNAs seen in Met 3 is highly significant relative to all the other samples (p = < 0.0001).

### 3.6 ERα associates with other Pol III transcribed genes

**Fig. 8:**
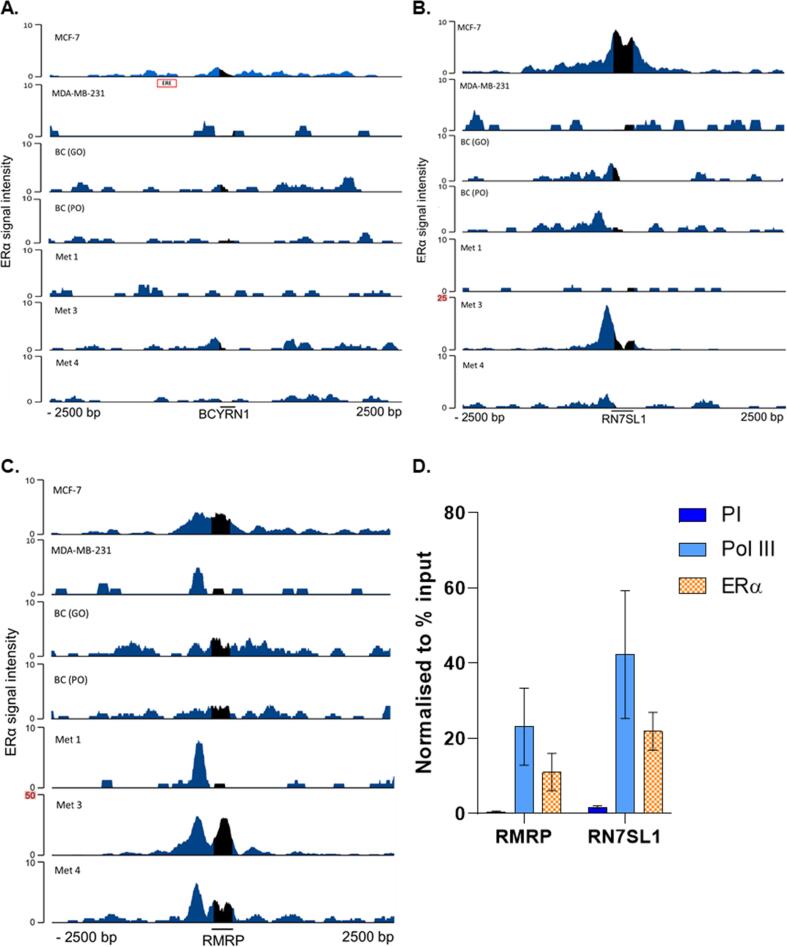
**ERα associates with the *RN7SL1* and *RMRP* genes in MCF-7 cells and breast cancer biopsies.** ERα binding intensity at BCYRN1 (A), RN7SL1 (B) and RMRP (C) in MCF-7 cells, MDA-MB-231 cells stably expressing ERα, good outcome (GO) or poor outcome (PO) breast cancer (BC) primary tumour samples and distant metastases (Met 1, 3 & 4). Window represents the ± 2.5 kb region from the centre of the genes. Red box highlights the approximate location of a potential ERE upstream of BCYRN1. Note difference in scale for Met 3 (highlighted). (D) ChIP-qPCR assays carried out at the RMRP and RN7SL1 genomic loci to confirm pol III and ERα enrichment. N = 3. Error bars are ± SEM. qPCR values have been normalised to % input.

BC200 (*BCYRN1*) is a long non-coding RNA that is synthesized by pol III. Predominantly expressed in the brain, it has been implicated as a regulator of protein synthesis (Samson et al., 2018). BC200 levels are aberrantly elevated in several types of malignancy (Chen et al., 1997, Samson et al., 2018); these include breast cancer, where high BC200 expression is associated with advanced stages of ductal carcinoma in situ (Chen et al., 1997, Iacoangeli et al., 2004, Singh et al., 2016). Manipulation of BC200 in MCF-7 cells revealed an oncogenic role through modulating the alternative splicing of Bcl-x and thus inhibiting apoptosis (Singh et al., 2016). Analysis of the BC200 promoter revealed an ERE-like sequence located 585 bp upstream of the transcriptional start site, that was reported to have enriched ERα binding in MCF-7 cells (Singh et al., 2016). In contrast, the ENCODE ERα ChIP-seq data showed no enrichment of ERα binding within a 2.5 kb window of the gene start site (Fig. 8A). The reason for this discrepancy is unclear, but might reflect the considerable heterogeneity that has been reported between MCF-7 cells from different sources (Ben-David et al., 2018). Analysis of clinical samples from primary tumours and metastases also provided minimal evidence for targeting of the BC200 promoter region.

7SL RNA has been shown to be overexpressed in breast tumours (Chen et al., 1997). In addition to its principal function in intracellular trafficking as the scaffold of the signal recognition particle (Walter and Blobel, 1982), it has been implicated in driving inflammatory responses in breast cancer (Nabet et al., 2017). Following exosome delivery from stromal to malignant cells, 7SL RNA activates the pattern recognition receptor RIG-1, which induces immune cell infiltration and influences tumour growth, metastasis and also therapy resistance (Nabet et al., 2017). 7SL RNA has also been shown to hybridise with mRNA encoding p53, thereby suppressing its translation (Abdelmohsen et al., 2014). The RN7SL1 gene has not, to our knowledge, been reported as estrogen responsive, but we found strong enrichment of ERα at this locus in MCF-7 cells and the metastatic sample Met 3 (Fig. 8B). ChIP-qPCR analysis confirmed ERα recruitment to the *RN7SL1* gene in MCF-7 cells (Fig. 8D) The promoter of *RN7SL1* includes sequences upstream of the transcription start site, as well as A- and B-box motifs within the transcribed region; ERα reads were evident in both these areas in MCF-7 cells, whereas ERα is localized to the upstream region in Met 3 and, much more weakly, in the primary tumour biopsies; the significance of this difference is unclear. No enrichment of ERα was observed at *RN7SL1* in Met 1 and MDA-MB-231 cells expressing the exogenous receptor. These data suggest significant heterogeneity in the interaction of ERα with the *RN7SL1* gene in breast cancer cells.

*RMRP* is a 267 nucleotide ncRNA involved in rRNA processing, which also associates with the telomerase catalytic subunit to produce an RNA-dependent RNA polymerase (Maida et al., 2009). It has been shown to promote cell cycle progression and proliferation (Goldfarb and Cech, 2017, Thiel et al., 2005, Vakkilainen et al., 2019). Germline RMRP mutations cause the inherited syndrome cartilage-hair hypoplasia (Ridanpää et al., 2001). Somatic mutations in the *RMRP* promoter lead to elevated expression in breast cancers and the locus also undergoes focal amplification in several tumour types, including breast (Rheinbay et al., 2020, Rheinbay et al., 2017). The RMRP promoter is located upstream of the transcription start site and showed interactions with ERα in MCF-7 cells, primary breast tumours and metastases, especially Met 3 (Fig. 8C). ChIP-qPCR analysis also shows strong ERα enrichment at the *RMRP* locus, relative to a pre-immune control (Fig. 8D). In contrast to other pol III promoters examined, some binding of exogenous ERα at the *RMRP* promoter was also detected in MDA-MB-231. Thus, the *RMRP* promoter is targeted by ERα, to a greater or lesser extent, in every case we have examined.

## 4 Discussion

Estradiol has been shown to rapidly increase transcription of rRNA and most tRNA genes in MCF-7 cells (Hah et al., 2011). Whether or not the Pol III-transcribed genes are targeted directly by ERα was not established, with the exception of tRNALeu and 5S rRNA genes (Fang et al., 2017, Zhong et al., 2014). Our analyses of two orthogonal MCF-7 ChIP-seq datasets provide evidence that endogenous ERα is attracted to a large proportion of tDNAs throughout the genome. This was unexpected, as consensus ERE motifs are not enriched in the vicinity of tDNAs and there is little evidence to date that tDNAs can be regulated from distal sites. Our findings are not limited to the MCF-7 cell line model, as ERα is also attracted to substantial numbers of tDNAs in ZR-75–1 cells, breast tumours and metastases. These data expand the repertoire of ERα targets to include some of the most highly expressed genes in cells. Indeed, tRNAs and 7SL RNA are the most abundant transcripts after rRNAs (Boivin et al., 2018), which are also induced by estrogen in MCF-7 cells (Hah et al., 2011). Thus, the well-characterized changes in mRNA expression that are triggered by ERα occur alongside regulation of the most abundant non-coding RNAs, which may impact heavily on capacity for mass accumulation and growth.

Motif analysis of the top ERα-bound tRNA genes did not detect canonical ERE or even half ERE sequences within 40 kb windows centred on the tDNAs. The possibility remains of degenerate or non-canonical motifs that are recognised directly by ERα. Despite some well-characterized exceptions, in the majority of cases of estrogen-responsive protein-coding genes ERα acts at a distance after binding to remote enhancer sites, rather than targeting proximal promoters (Fullwood et al., 2009). Our data do not address this scenario. Although ChIP-seq signals are seen for ERα at or near many tDNAs without consensus ERE motifs, these peaks might potentially reflect crosslinking of ERα that has bound to distal enhancers and then accessed tDNAs through long-range looping. Precedent for such control comes from an insightful study of macrophage differentiation, where changes in remote interactions correlate with variations in transcription factor binding to specific tRNA genes (Van Bortle et al., 2017). However, causal relationships were not established for these correlations and the authors concluded that tDNA regulation is most likely to be controlled predominantly by proximal binding (Van Bortle et al., 2017).

ERα can also be brought to tDNAs through interactions with other proteins, rather than direct recognition of DNA sequences, and this has been estimated to account for ∼ 10% of its binding events (Stender et al., 2010). At pol III-transcribed genes, this might be achieved through association with TFIIIB, which coimmunoprecipitates with ERα from MCF-7 cells (Fang et al., 2017). TFIIIB binds adjacently to the transcription start site and recruits pol III to its templates, a function that is essential for tRNA synthesis (Kassavetis et al., 1990). Its location is consistent with the peak of ERα in heatmaps and plots of average signal intensity (Fig. 2A and C). Analysis of binding patterns at individual genes also supports colocalization of ERα with TFIIIB at the *tRNA-Arg-CCG-2*–*1* and *tRNA-Met-CAT-1*–*1* genes (Fig. 2D), as well as *RN7SL1* and *RMRP* ((Fig. 8B and 8C). However, the ERα peak at the *tRNA-Pro-TGG-1*–*1* and *tRNA-Leu-AAG-2*–*4* genes is downstream of where TFIIIB is expected (Fig. 2D). One or more alternative factors might recruit ERα to these more downstream sites at *tRNA-Pro-TGG-1*–*1* and *tRNA-Leu-AAG-2*–*4*.

As TFIIIB is required for expression of all pol III-transcribed genes, it is predicted to be available for exogenous ERα to access at active tDNAs in MDA-MB-231 cells and yet no interaction was detected at these loci (Fig. 5B and 5C). Examination of pol II-transcribed ERα targets shows that this was not due to inactivity of the exogenous receptor (Fig. 5A and (Stender et al., 2010). A possibility that cannot be excluded is that detection by ChIP is prevented in some contexts by inaccessibility to crosslinking or antibody. Alternatively, the post-translational modification state of TFIIIB and/or ERα in MDA-MB-231 cells might preclude their stable interaction. One or more additional factors might also be necessary for ERα recruitment to tDNAs and availability of these might be more restricted than that of the ubiquitous TFIIIB. Involvement of additional factors is consistent with loci such as *tRNA-Leu-AAG-2*–*4* (Fig. 2D), where ERα peaks at sites other than immediately upstream of the start site where TFIIIB is always positioned. The role and identity of such putative factors, potentially involved in recruiting ERα to tDNAs, will require further investigation.

Even in MCF-7 cells, the ChIP-seq signal intensity for ERα at tDNAs is generally much lower than that seen for well-established targets such as GREB1. Several possibilities might account for this difference. For example, tethering through other proteins might lower the efficiency of ERα crosslinking to tDNAs. Additional components of transcription factor complexes could restrict antibody accessibility and thereby reduce ChIP efficiency. In principle, such confounding factors may contribute to the low signal intensities on most tDNAs and explain why they have not been identified previously as ERα targets. It may also be that ERα is attracted to tDNAs primarily by their accessibility in chromatin and is often rapidly released unless captured by other proteins. Binding that is weak and/or transient may nevertheless have functional impact, as demonstrated by the robust transcriptional induction of these genes by oestradiol (Hah et al., 2011).

The Brf1 gene encodes an essential subunit of TFIIIB and is itself bound and induced by ERα in MCF-7 cells (Fang et al., 2017). As alcohol increases the risk of breast cancer, it is noteworthy that expression of BRF1 and tRNA is increased by alcohol in cell lines, mouse models and humans (Zhong et al., 2016, Zhong et al., 2011). Furthermore, the ability of alcohol to promote colony formation by breast or liver cells can be blunted by specifically blocking the induction of BRF1, implying that normal BRF1 levels are insufficient for the transforming effect of alcohol in these models (Fang et al., 2017, Yi et al., 2017, Zhang et al., 2013).

Abnormally elevated tRNA levels have been documented in breast cancers by several studies (Krishnan et al., 2016, Pavon-Eternod et al., 2009, Zhang et al., 2018). Direct induction by ERα, as well as induction of the Brf1 gene, are likely to contribute to this overexpression, but additional molecular mechanisms are also well-documented (Grewal, 2015, White, 2005). These include transcriptional activation by Ras (Johnson et al., 2000) and MYC (Gomez-Roman et al., 2003), as well as loss of tRNA gene repression by the RB (White et al., 1996), p53 (Cairns and White, 1998), PTEN (Woiwode et al., 2008) and BRCA1 (Veras et al., 2009) tumour suppressors. Furthermore, progesterone receptor has been shown to bind pol III and localise at tRNA genes in patient-derived xenograft models (Finlay-Schultz et al., 2017). A multitude of changes to the regulatory landscape in breast cancers is expected to impact on the production of noncoding RNAs by pol III.

Abnormal expression of tRNAs is likely to have functional impact. Elevated levels of 14 tRNAs are significantly associated with poor prognosis in breast cancer (Krishnan et al., 2016) and specific tRNAs have been shown to promote proliferation (Kwon et al., 2018, Pavon-Eternod et al., 2013, Wang et al., 2018) and/or metastasis (Birch et al., 2016, Clarke et al., 2016, Goodarzi et al., 2016) in a variety of systems. For example, overexpression of tRNA-Arg-CCG was shown to promote invasion by breast cancer cells *in vitro* and metastasis in mouse models through codon-dependent effects on stability and translation of specific pro-metastatic mRNAs (Goodarzi et al., 2016); it is therefore noteworthy that the tRNA-Arg-CCG-2–1 gene was targeted strongly in the MCF-7 dataset. Proliferative induction has been documented extensively for RMRP (Goldfarb and Cech, 2017, Thiel et al., 2005, Vakkilainen et al., 2019), which has been identified as an oncogenic driver of breast cancer through large unbiased screens (Rheinbay et al., 2020, Rheinbay et al., 2017). 7SL RNA is highly enriched in exosomes circulating in serum of breast cancer patients and promotes tumour growth by triggering inflammatory signalling (Nabet et al., 2017). These observations establish the potential importance of our discovery that *RN7SL1*, *RMRP* and hundreds of tDNAs are targeted by ERα in breast cancer tumours and metastases.

## CRediT authorship contribution statement

**Jodie R. Malcolm:** Methodology, Investigation, Writing – original draft, Visualization. **Natasha K. Leese:** Methodology, Investigation, Visualization. **Philippa I. Lamond-Warner:** Methodology, Investigation, Visualization. **William J. Brackenbury:** Supervision. **Robert J. White:** Supervision, Conceptualization, Writing – review & editing.

## Declaration of Competing Interest

The authors declare that they have no known competing financial interests or personal relationships that could have appeared to influence the work reported in this paper.
