# Supplementary material for: Widespread association of ERα with RMRP and tRNA genes in MCF-7 cells and breast cancers
Source: Gene. 2022 May 5;821:None. doi: 10.1016/j.gene.2022.146280 (PMC8942118; doi:10.1016/j.gene.2022.146280)
Supplement: Supplementary data 1 [file mmc1.docx]

**SUPPLEMENTARY DATA**

| **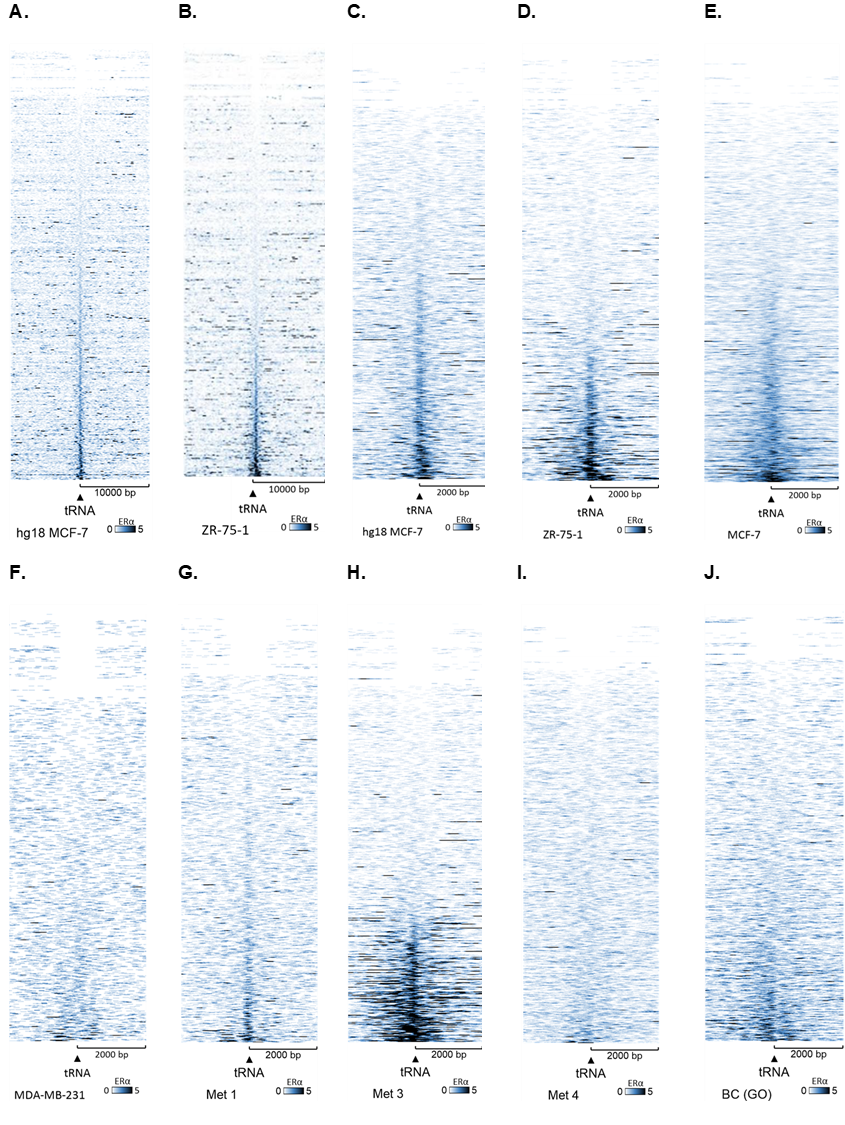** |
| --- |
| **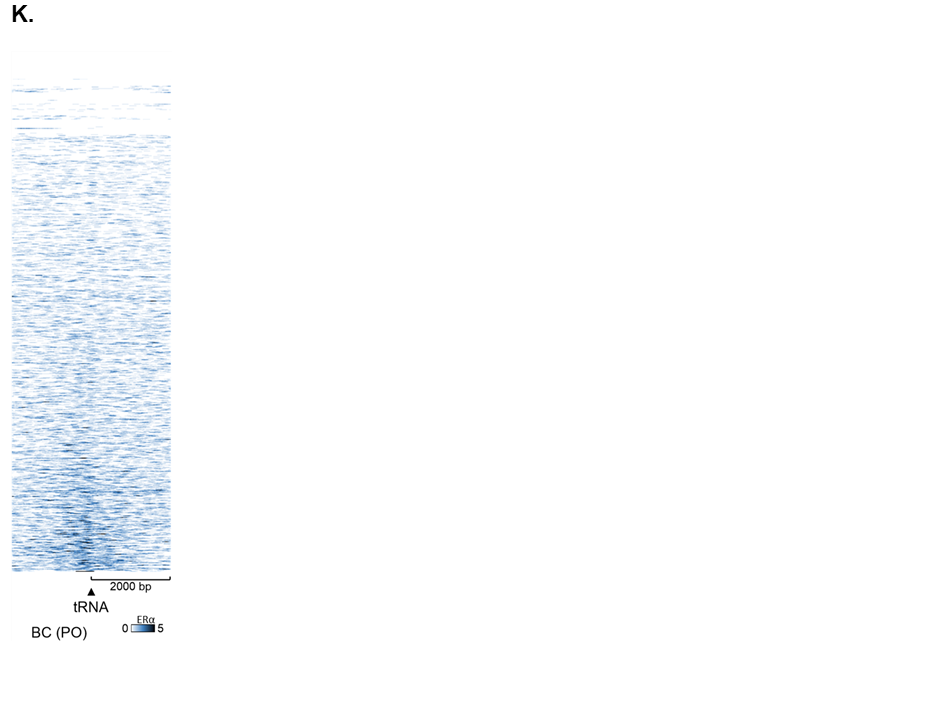** |
| **Supplementary Figure S1.** (A) Heatmap of ERα binding events across hg18 tRNA genes in the MCF-7 cell line (NCBI SRA). Window represents the ± 10 kb region from the centre of tRNA genes. Genes arranged in order of increasing Q-value. (B) Heatmap of ERα binding events across hg18 tRNA genes in the ZR-75-1 cell line (NCBI SRA). Window represents the ± 10 kb region from the centre of tRNA genes. Genes arranged in order of increasing Q-value. (C-K) Heatmaps of ERα binding events in the MCF-7 (NCBI SRA; C), ZR-75-1 (D), MCF-7 (ENCODE; E), MDA-MB-231 (F), Met 1 (G), Met 3 (H), Met 4 (I), BC GO (J) and BC PO (K) ChIP-seq data sets. Window represents the ± 2 kb region from the centre of tRNA genes. Genes arranged in order of increasing Q-value |

| **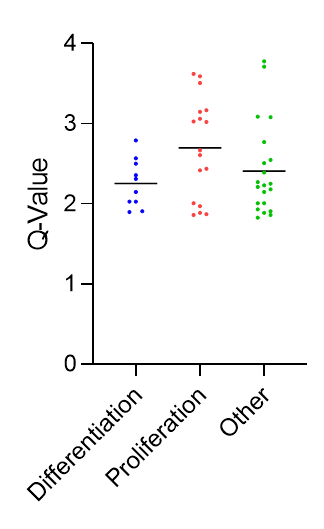** |
| --- |
| **Supplementary Figure S2.** Quantification of ERα binding at the top twenty-five ERα-bound tRNA genes based on their preferential induction during differentiation, proliferation, or other cellular processes. Outlier values omitted. |

| **Supplementary Table ST1. The top 50 ERα-bound hg38 tRNA genes in MCF-7 cells, identified through ChIP-seq analysis.** |
| --- |
| **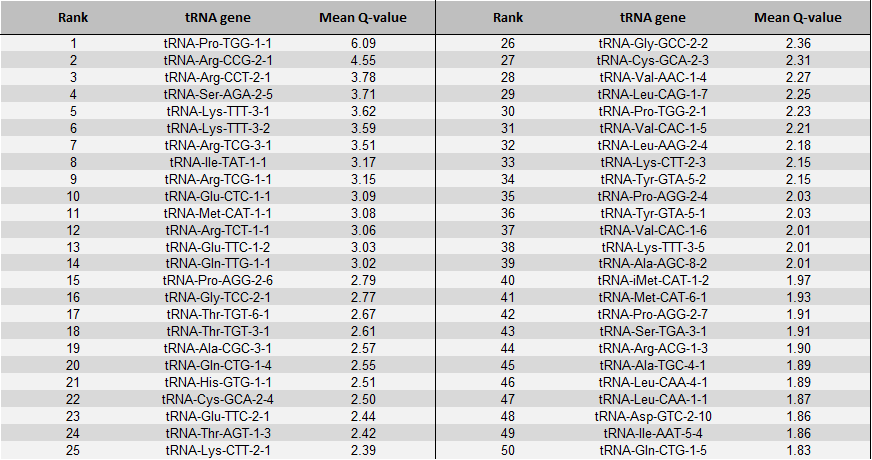** |

| **Supplementary Table ST2. Descriptive statistics of ERα Q-values (indicative of binding at tRNA genes) in MCF-7 cells, good outcome (GO) or poor outcome (PO) breast cancer (BC) primary tumour samples and distant metastases (Met 1, 3 & 4).** |
| --- |
| **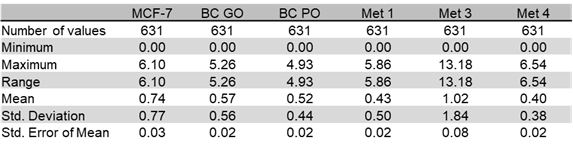** |
